# Sirolimus as Rescue Therapy for Refractory/Relapsed Immune Thrombocytopenia: Results of a Single-Center, Prospective, Single-Arm Study

**DOI:** 10.3389/fmed.2020.00110

**Published:** 2020-03-31

**Authors:** Yimei Feng, Yunshuo Xiao, Hongju Yan, Ping Wang, Wen Zhu, Kaniel Cassady, Zhongmin Zou, Kaifa Wang, Ting Chen, Yao Quan, Zheng Wang, Shijie Yang, Rui Wang, Xiaoping Li, Lei Gao, Cheng Zhang, Yao Liu, Peiyan Kong, Li Gao, Xi Zhang

**Affiliations:** ^1^Medical Center of Hematology, The Xinqiao Hospital of Third Military Medical University, Chongqing, China; ^2^State Key Laboratory of Trauma, Burns and Combined Injury, Third Military Medical University, Chongqing, China; ^3^Irell and Manella Graduate School of Biological Sciences of City of Hope, Duarte, CA, United States; ^4^Department of Chemical Defense, School of Preventive Medicine, Third Military Medical University, Chongqing, China; ^5^School of Mathematics and Statistics, Southwest University, Chongqing, China

**Keywords:** sirolimus, mTOR, refractory/relapsed immune thrombocytopenia, peripheral tolerance, lymphocyte subsets

## Abstract

Immune thrombocytopenia (ITP) is an autoimmune disease which arises due to self-destruction of circulating platelets. Failure to respond or maintain a response to first-line treatment can lead to refractory/relapsed (R/R) ITP. The mechanism remains complicated and lacks a standard clinical treatment. Sirolimus (SRL) is a mammalian target of rapamycin (mTOR) inhibitor that has been demonstrated to inhibit lymphocyte activity, indicating potential for SRL in treatment of ITP. Activation of the mTOR pathway in autoimmune diseases suggests that SRL might be a useful agent for treating ITP. Accordingly, we initiated an open-label, prospective clinical trial using SRL for patients with R/R ITP (ChiCTR-ONC-17012126). The trial enrolled 86 patients, each dosed with 2–4 mg/day of SRL. By the third month, 40% of patients (34 of 86) achieved complete remission (CR) and 45% of patients (39 of 86) achieved partial remission (PR), whereby establishing an overall response rate (ORR) of 85%. By 6 months of treatment, 41% of patients (32 of 78) achieved CR and 29% of patients (23 of 78) achieved PR, establishing an ORR of 70% without serious side effects. After 12 months follow-up, the ORR remained at 65%. We also found that SRL treatment exhibited higher efficacy in achieving CR in ITP patients who were younger than 40 years old or steroid dependent by univariate analysis. Importantly, in patients who responded, SRL treatment was associated with a reduction in the percentage of Th2, Th17 cells, and increase in the percentage of M-MDSCs and Tregs, indicating that SRL may reestablish peripheral tolerance. Taken together, Sirolimus demonstrated efficacy as a second-line agent for R/R ITP.

## Introduction

Immune thrombocytopenia (ITP) is a complicated autoimmune disease characterized by low platelet counts. First-line treatment regimens are administered to inhibit autoantibody production and platelet degradation, and consist of treatments corticosteroids, such as prednisone 1 mg/kg/d for 2–4 weeks, followed by tapering. Splenectomy, rituximab and thrombopoietin receptor agonists (TPO-RAs, Eltrompobag and romiplostim) are commonly used as second-line therapies and are curative for some patients; however, response rates among individuals vary greatly. To date, refractory/relapsed (R/R) ITP remains under-treated for many patients (1) However, about 20–30% of ITP patients show no, or poor response to both first-line and second-line therapies, especially for those diagnosed as R/R ITP. The mechanism by which R/R ITP develops remains complicated and lacks a standard clinical treatment. Thus, establishing a standard treatment for these difficult-to-treat patients remains of critical need in the clinic.

Sirolimus (SRL) is a mammalian target of rapamycin (mTOR) inhibitor that has been shown to induce apoptosis of both activated and irregular lymphocytes to promote tolerance (2, 3). Consequently, SRL has emerged as one of the most promising immunosuppressive agents after transplantation of either solid organs or allogeneic stem cells (4). Case reports and small case series have suggested that SRL is effective for the treatment of both autoimmune lymphoproliferative syndrome (ALPS) (5) and autoimmune cytopenias (6). Activation of mTOR is a hallmark of and biomarker in autoimmune diseases, such as systemic lupus erythematosis (SLE) (7–9) and ITP (10). Importantly, sirolimus was shown to improve thrombocytopenia in SLE (11). Jasinski et al. (12) treated 12 refractory ITP patients with SRL, resulting in 73% of the patients achieving CR by 3 months after treatment. Based on these reports, we initiated an open-label prospective clinical trial using SRL for patients with R/R ITP. Our results suggest that SRL is an effective and well-tolerated option for achieving acceptable clinical efficacy in this patient population. We found that SRL could modulate T, B, and myeloid-cell subsets, suggesting that SRL may maintain or establish peripheral tolerance in autoimmune ITP patients. Thus, we propose that SRL should be considered as a safe and effective second-line agent for the treatment of refractory/relapsed ITP.

## Patients and Methods

This prospective single-arm study was conducted between January 2016 and January 2019 at our center (ChiCTR-ONC-17012126). According to our calculated sample size, 86 R/R ITP patients with platelet count <50 × 10^9^/L were ultimately enrolled into this clinical trial. Inclusion criteria required the patients to have failed corticosteroid therapy (prednisone, methylprednisolone or dexamethasone) and at least one 2nd-line agent, including TPO, cyclosporine, splenectomy, rituximab or traditional Chinese medicine (TCM). SRL was given at a dose of 2 mg/day orally (maximum initial dose of 4 mg/day) and targeted a plasma concentration of 5~15 ng/ml in circulation. The anticipated length of treatment with SRL was 3 months, then tapered down 0.5 mg/2 weeks. Patients received follow-up care for 12 months. Platelet count was measured once every 2 weeks. Complete response (CR) was defined as platelet count >100 × 10^9^/L, and partial response (PR) as platelet count >30 × 10^9^/L and at least a 2-fold increase in the baseline count (13). Main exclusion criteria were: previous treatment with mTOR inhibitors; any other present malignancy; any other concomitant serious illness or organ system dysfunction; active hepatitis B or C infection; HIV infection; pregnant or lactating women. The main removal protocol included: patients with intolerable serious adverse events; the disease progressed under the trial protocol; patient requested to quit; the patients' condition changed or died, which were not related to the experimental factors, during the treatment and follow-up period.

**Figure 1:**
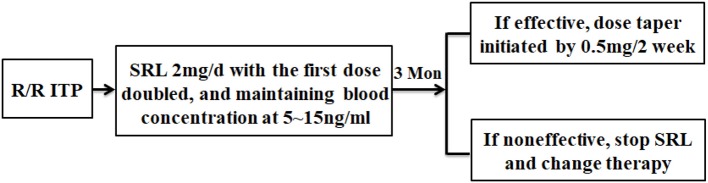
The flowchart of this study. R/R ITP patients who met the inclusion criteria were administered SRL 2 mg/d, then the dose was adjusted to maintain blood concentration between 5~15 ng/ml. After 3 months of SRL oral administration, evaluation of SRL efficacy was conducted. Responders started to taper their dose by 0.5 mg/2 week. Some patients had the tendency of thrombocytopenia recurrence during tapering and, in this situation, treatment with very low SRL doses was required for 6 months or more. The non-responders stopped SRL and received other therapy.

We collected data including age-at-diagnosis, sex, first and second-line therapies used, platelet count at the initiation of SRL and at the 3-, 6-, and 12-month time points of SRL therapy, total duration of SRL therapy, and adverse events (according to CTCAE version 4.0) (Figure 1). This study was approved by the ethics committees of Xinqiao Hospital and required that all human participants provide written informed consent.

### Flow Cytometry

Peripheral blood samples were collected at the following points: (1) before treatment in ITP patients; (2) after treatment in patients who obtained complete response and partial response; (3) after treatment in patients who did not respond to Sirolimus. Flow cytometry to characterize T, B and myeloid lymphocytes was performed for each patient. Th2 cells were defined as CD45^+^CD3^+^CD4^+^CD183^−^CD196^−^, M-MDSCs cells were defined by CD45^+^DR^low/−^CD33^+^CD11b^+^CD14^+^CD3^−^CD56^−^, and activated Th17 cells were defined by CD45^+^CD3^+^CD4^+^CD62L^+^Rorgt^+^. Cells were analyzed by a ten color-flow cytometry (BD FACSCanto II). Total events of 50,000 were gated for downstream analysis based on forward (FSC) and side-scatter (SSC) characteristics.

### Statistical Analysis

When design this trial, according to the literatures, the remission rate of the control second-line treatment was set to 60%, and the estimated response rate of the experimental group was to be 80% (12, 14). We used a professional sample calculation formula, chose α = 0.05; power = 0.8; β = 0.2 and calculated the sample size as 78 cases. The drop-out rate was set to 10%, resulting in the final patient number as 86. The primary endpoint was overall response rate after SRL treatment. The secondary endpoints included the time of SRL treatment required to demonstrate efficacy and minimal adverse events. Continuous variables are expressed as mean ± SDs. One-way ANOVA test was performed to the level of lymphocyte subsets between the different groups, or during different phases of treatment and follow-up. The Chi-square test was used to analyze the intergroup difference as univariate analysis, and Logistic regression (enter method) was used to evaluate the risk factors affecting efficacy as multivariate analysis. All data were analyzed and visualized by SPSS 19 and Graphpad Prism 8. Results were considered statistically significant when the *p*-value was < 0.05.

## Results

### Patient Characteristics

A total of 86 patients (22 males, 64 females) with refractory and relapsed ITP were included and treated with SRL. The median age of all included patients was 40 years (range, 5–85 years), and the median time from first diagnosis to enrollment was 12 months (range, 1–492 months). The time from diagnosis to enrollment of 44 patients (51%) was shorter than 12 months and the rest 42 patients (49%) belonged to chronic ITP, whose course of disease were longer than 12 months. All patients had failed 1 to 5 of the first- and second-line therapies and included steroid-resistant (42%, 36 patients), steroid-dependent (30%, 26 patients, who were undergoing steroid tapering and achieved a platelet count of 30~50 × 10^9^/L), and steroid plus rituximab /cyclosporine /splenectomy or /TCM (27%, 24 patients). In detail, 62 patients (72%) were administrated with SRL as the second-line therapy, including steroid resistant and dependence. Among those patients who were failed to steroid therapy and had not received sirolimus before, two patients accepted splenectomy, eight patients had cyclosporine therapy, six patients have been treated with rituximab, eight patients accepted TCM therapy. Besides, 17 patients in this cohort study received TPO agent additionally, including the recombinant human thrombopoietin (Chinese Shenyang biologics agent) and recombinant human interleukin 11 before enrolled, however, the clinical effect was poor (Table 1).

**Table 1:** Patient characteristics.

**Patient characteristics**	**Case(*n* = 86)**	**Rate**
**Age (years)**
≤ 40y	43	50%
>40y	43	50%
**Gender**
Male	22	26%
Female	64	74%
**Time from diagnosis to enrollment**
<12 mon	44	51%
≥12 mon	42	49%
**Previous therapy**
Steroid-resistant	36	42%
Steroid-dependence	26	30%
Steroid plus Splenectomy	2	2%
Steroid plus Cyclosporine	8	9%
Steroid plus Rituximab	6	7%
Steroid plus TCM	8	9%
**Platelet count**		
<30 × 109/L	60	70%
≥30–50 × 109/L	26	30%

### Efficacy

Three months after initiation of SRL treatment in the ITP cohort, 40% of patients (34 of 86) achieved CR and 45% (39 of 86) achieved PR. The overall response rate (ORR,CR+PR) reached 85% while 15% patients were non-responders (NR,13 of 86).

**Figure 2:**
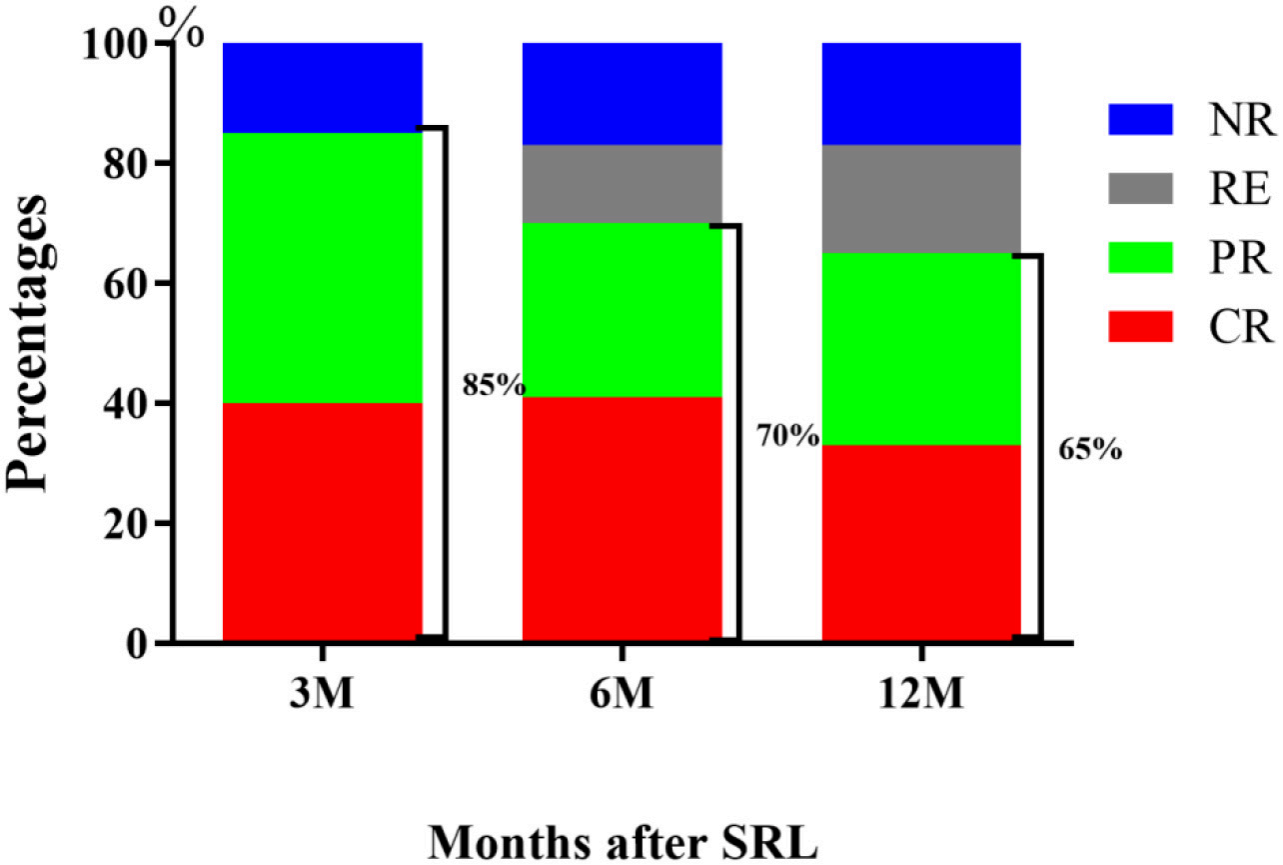
Response of R/R ITP to SRL. The outcome demonstrated that SRL was effective for R/R ITP, with an ORR at 3 months of 85%, 6 months of 70 and 65% at 12 months.

Throughout the 6 month treatment period, 8 patients were lost to follow-up, 41% achieved CR (32 of 78 patients) and 29% achieved PR (23 of 78 patients). The ORR decreased from 85% at the 3 month period to 70% by the 6 month following treatment. Of note, the CR rate remained unchanged, but several of the patients who previously achieved PR by the 3 month mark relapsed. After 12 month follow-up, the CR rate was 33% (26/78), with PR 32% (25/78). The ORR decreased to 65%, because 4 patients relapsed, of whom achieved CR by the 6 month mark (Table 2, Figure 2).

**Table 2:** Clinical outcomes after SRL treatment.

**Therapeutic effect at 3 months (*****n*** **=** **86)**		
	**Case**	**Rate**
ORR (CR + PR)	73	85%
CR	34	40%
PR	39	45%
NR	13	15%
**Therapeutic effect at 6 months (*****n*** **=** **78)**		
ORR (CR + PR)	55	70%
CR	32	41%
PR	23	29%
RE	10	13%
NR	13	17%
**Therapeutic effect at 12 months (*****n*** **=** **78)**		
ORR (CR + PR)	51	65%
CR	26	33%
PR	25	32%
RE	14	18%
NR	13	17%

Secondly, we compared the efficacy of SRL from the following five subgroups: (1) age (≤ 40y vs. >40y); (2) gender; (3) time from diagnosis to enrollment (<12 mon vs. ≥12 mon); (4) SRL used as second-line or multiple-line treatment, and (5) the platelet count at time of treatment (<30 × 10^9^/L and 30–50 × 10^9^/L). Univariate analysis showed that age and PLT number had influences on SRL effect. The CR rate of patients under 40 years old was higher than those over 40 years old (60 Vs. 37%, *p* = 0.031). Additionally, patients with steroid dependence were more likely to achieve at CR (63 Vs. 43%, *p* = 0.039), whose PLT number were between 30 and 50 × 10^9^/L. However, multivariate analysis showed there was no statistical difference in CR rate between the five subgroups (Table 3).

**Table 3:** Patient subset analysis of CR rate 3 months after treatment.

**Prognostic variables**	**CR rate**	**Univariate analysis**		**Multivariate analysis**	
		***P*-value**	**HR (95% CI)**	***P*-value**	**HR (95% CI)**
**Age**
≤ 40y	60%	0.031	2.593 (1.084–6.201)	0.135	0.498 (0.200–1.241)
>40y	37%				
**Gender**
Male	45%	0.908	1.059 (0.400–2.799)	0.839	1.119 (0.377–3.321)
Female	47%				
**Course of disease**
<12 mon	41%	0.353	1.500 (0.637–3.534)	0.170	1.976 (0.377–3.321)
≥12 mon	51%				
**Sirolimus treat**
As the second line	48%	0.575	1.313 (0.506–3.401)	0.508	0.697 (0.240–2.030)
As the multiple line	42%				
**Platelet count**
<30 × 10^9^/L	43%	0.039	2.661 (1.039–6.813)	0.055	2.750 (0.978–7.734)
≥30 × 10^9^/L (30–50)	63%				

**Figure 3:**
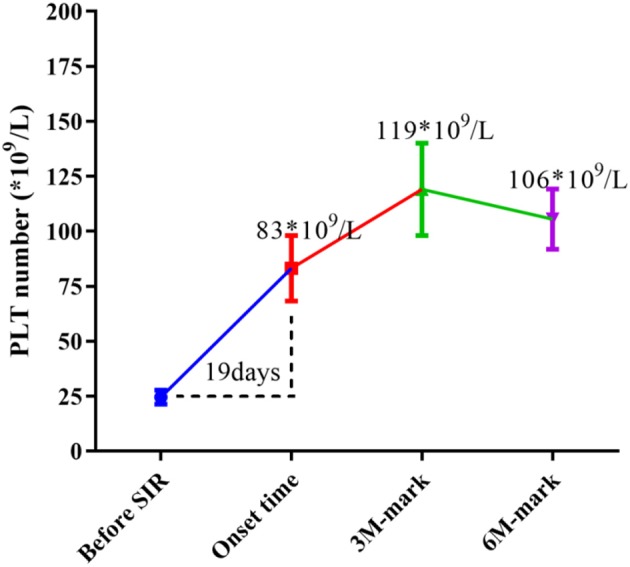
Changes in median PLT numbers during SRL treatment. Before SRL treatment, the average number of PLTs per patient was 25 × 10^9^/L (as baseline), and the median period for SRL to take effect was 19 days, when the average number of PLT of patients increased to 83 × 10^9^/L (*p* < 0.0001 vs. baseline). Mean PLT counts increased to an average of 119 × 10^9^/L by 3 month-mark, and maintained an average of 106 × 10^9^/L by 6 month-mark (*p* < 0.0001 vs. baseline).

At enrollment, the average number of PLTs per patient was 25 × 10^9^/L, and the median period for SRL to take effect was 19 days (range 5~110 days), when the average number of PLTs/patient increased to 83 × 10^9^/L(*p* < 0.0001 vs. baseline). Mean PLT counts increased to an average of 119 × 10^9^/L by 3 months after treatment, and maintained an average of 106 × 10^9^/L by 6 month following treatment in those patients who responded to SRL (*p* < 0.0001 vs. baseline) (Figure 3).

### Toxicity/Safety

Adverse events presented mainly as grade 1, 2 severity (Table 4). The incidence of dyslipidemia was as high as 12%, including increased triglyceride and lipoprotein. Five patients (6%) with increased serum levels of alanine aminotransferase (ALT) and aspartate aminotransferase (AST) were recorded. Six patients (7%) exhibited canker sores, which healed after Vitamin B administration. Skin rash (four patients, 5%), arthralgia (two patients, 2%), and edema of lower extremity (two patients, 2%) were also documented. One patient developed severe interstitial pneumonia, a grade 3 adverse event, but recovered after responding to anti-infective therapy (Table 4). Although SRL has been reported to induce leukopenia and hemocytopenia, we did not observe sirolimus to induce leukopenia or hemocytopenia. This is likely because the blood concentration of SRL was kept at 5–15 ng/ml in our study, which help ensure low drug toxicity.

**Table 4:** Adverse events.

**Adverse events**	**Cases(*n* = 86)**	**Rate**
**Dyslipidemia**
Grade-1	9	10%
Grade-2	2	2%
**High aminotransferase**
Grade-1	5	6%
**Canker sores**
Grade-1	4	5%
Grade-2	2	2%
**Skin rash**
Grade-1	4	5%
**Arthralgia**
Grade-1	1	1%
Grade-2	1	1%
**Edema of lower extremities**
Grade-1	2	2%
**Interstitial pneumonia**
Grade-3	1	1%

### Lymphocyte Subset Analysis

**Figure 4:**
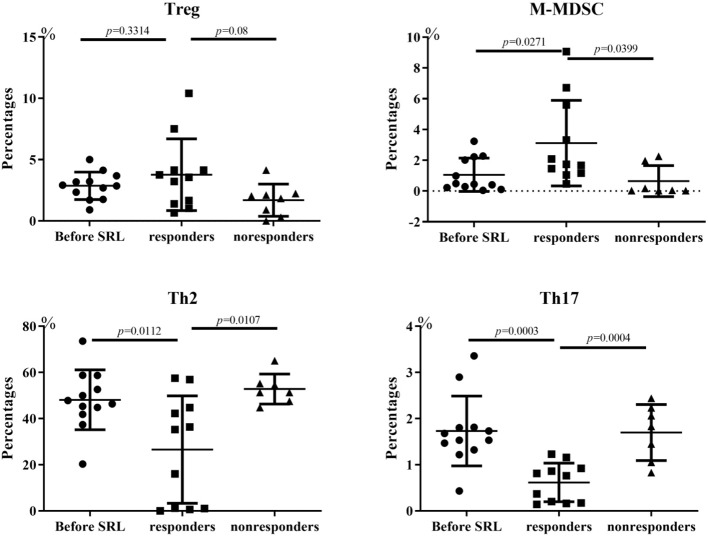
Changes of lymphocyte subsets of ITP patients. Before SRL administration, the proportion of Treg cells was lower in the periphery; however, after SRL intervention, Treg cells in patients who responded were elevated than that in pre-treatment (*p* = 0.3314) and non-responders (*p* = 0.08). The changes of M-MDSC is similar with Treg cell trend, with statistical difference (*p* = 0.0271 Vs. pre-treatment; *p* = 0.0399 Vs. non-responders). In contrast, SRL treatment alleviated both Th2 and active Th17 cell ratios in ITP patients dramatically (*p* < 0.05). One-way ANOVA test was performed to the level of lymphocyte subsets between the different groups.

Flow cytometry analysis was performed to measure the lymphocyte subsets in three groups: ITP patients prior to SRL treatment, ITP patients who responded to SRL and ITP patients who did not respond to treatment with SRL. We measured subsets in some of the patients and observed a significant change in the proportions of T or B subsets before and after SRL treatment. Both Th2 and activated Th17 cell percentages in ITP patients were increased in the periphery before SRL treatment; however, after SRL administration, these cell populations decreased dramatically. In contrast, before SRL administration, the proportion of M-MDSC and Treg subsets were lower in the periphery; however, after SRL intervention, percentages of both of these tolerogenic subsets of cells in patients who responded were elevated than that in either non-responders or patients prior to treatment (Figure 4).

## Discussion

There is no standard of care for treating R/R ITP; current treatments are varied and include splenectomy or administration of cyclosporine, rituximab and TPO-RAs. In our report, we tested the effect of an mTOR inhibitor, sirolimus in the treatment of R/R ITP. We found that treatment with SRL led to an ORR of 85% at the third month after treatment, 70% at 6 months, and 65% at 12 months following treatment (Figure 2). These results indicate that an mTOR inhibitor may be effective as a treatment of R/R ITP. Additionally, although we found that sirolimus treatment was more effective in achieving CR in ITP patients who were younger than 40 years old or steroid dependent by univariate analysis, they did not reach significance by multivariate analysis, which may need more data to be collected for further confirmation (Table 3).

Splenectomy is reported as an effective treatment for R/R ITP. In one report, 1 year after splenectomy, 88.7% patients undergoing splenectomy had achieved CR (15). However, many patients with refractory and relapsed ITP refuse to undergo splenectomy (16), because surgery is invasive and potentially associated with infection and thrombosis. Rituximab is also used to treat ITP and is associated with response rates of 40~50%. In one clinical study, Tran et al. reported (17) the efficacy of rituximab treatment in refractory and relapsed ITP was 44% at the 8 weeks after infusion but failed to reach a primary endpoint of 50% response. Moreover, rituximab is associated with a series of adverse reactions such as hepatitis B virus activation, bacterial infection, hypogammaglobulinemia, and infusion reactions (18). TPO-RAs, such as Eltrompobag and romiplostim, have been shown to be efficacious and safe (19) in the treatment of ITP. In a study in China, 57.7% of Eltrompobag-treated chronic ITP patients could achieve platelet counts of ≥50 × 10^9^/L (20). However, in economically disadvantaged areas of southwest China, the high cost of TPO-RAs has limited their widespread application, thus requiring patients to turn to cheaper alternatives. SRL, an mTOR inhibitor, has been used broadly for immune suppression after organ transplantation or hematopoietic stem cell transplantation (21, 22), but few groups have reported its efficacy in ITP therapy (12). Activation of mTOR and efficacy of therapeutic blockade was previously demonstrated in thrombocytopenia secondary to SLE (11). Our present clinical trial demonstrated that SRL was effective for R/R ITP, with an ORR at 3 months of 85%, 6 months of 70 and 65% at 12 months. Importantly, adverse events were minor and SRL was well-tolerated by most patients. In our cohort study, four patients expressed dissatisfaction with the course of SRL therapy and elected to leave the study after achieving PR 3 months after treatment. Another two patients stopped taking SRL when they achieved CR 3 months after treatment, subsequently relapsed and left the study. Importantly, none of the patients who were responders withdrew from the study because of adverse events. Moreover, throughout the study, plasma drug concentration was closely monitored, which may account for the spectacular safety profile of SRL in our study. Together, SRL appears to be a safe and effective therapeutic option for R/R ITP patients.

Li et al. (23) demonstrated that treatment with rapamycin plus prednisone can increase PLT number in chronic ITP patients; increased PLT number was associated with a significant increase in Treg cell levels and TGF-beta in patient plasma after treatment. Consistent with this observation, levels of Treg cells were lower in ITP patients compared with healthy donors prior to treatment in our cohort study. Treatment with SRL was associated with an increased Treg level in patients who achieved CR and PR. It has been reported that impairment of Treg cells contributes to the development of ITP (24). Interestingly, Treg levels in non-responders trended lower than percentage of Tregs in responders; however, this trend was not significant (Figure 4). We hypothesize that in some patients, SRL treatment may reestablish normal levels of peripheral Tregs; however, this may not be sufficient to restore tolerance in some patients. M-MDSC regulation of ITP is unknown. Here, we hypothesize that dysregulation of M-MDSC might also contribute to the pathogenesis of ITP, as treatment with SRL was associated with a restoration of M-MDSC proportions. Together, we observed an increase in MDSC proportion emerging after SRL intervention, and we propose that both Tregs and MDSCs may play a critical role in the maintenance of peripheral tolerance by suppressing self-reactive lymphocytes. Further studies are required to test these hypotheses.

Previous studies have shown that Th cells (Th1, Th2, and Th17 cells) and their secreted cytokines are involved in ITP pathogenesis (25). Th1 cells mainly secrete IL-2, IFNγ, and TFNα, mediating cytotoxic response and local inflammatory response. Th2 cells mainly secreted IL-4, IL-6, and IL-10, stimulating B cell proliferation to produce antibodies (26). Levels of activated Th17 cells were reported to be elevated in ITP patients, and produced large amounts of IL-17, IL-21, and IL-22 (27, 28). In ITP, whether Th1 or Th2 subsets are dominant in mediating pathogenesis is still controversial. In our report, treatment with SRL was associated with a reduction in Th2 and Th17 cell levels, which may alleviate platelet destruction and help explain in part the mechanism of SRL treatment of ITP.

Our study was limited by its design as a single arm trial rather than a randomized controlled trial (RCT). At the time of study design, many of our patients had failed steroids, and were reluctant to accept splenectomy or TPO-RAs, out of financial difficulties or hesitation to undergo surgery. A proportion of enrolled patients (30%, 26/86) receiving SRL treatment were steroid-dependent and underwent steroid tapering while achieving a platelet count of 30–50 × 10^9^/L, and these steroid dependent patients were more likely to achieve CR after SRL treatment. These results may indicate a synergistic response of low-dose steroid and SRL; this observation is in agreement with the report that mTOR inhibitor plus low dose steroid could provide a new, promising option for therapy of ITP (23). For newly diagnosed ITP, will the addition of sirolimus as a first-line therapeutic along with low-dose steroids increase efficacy and reduce recurrence? Based our results, we believe it may be possible to use SRL as a first-line agent. This is largely due to the observation that the median period for SRL to take effect was 19 days, and there were less adverse events than in patients treated with steroids. This, of course, needs to be verified by randomized controlled trials. We have begun a clinical trial (ChiCTR1900020657) with 15 major ITP treatment centers in China, testing newly diagnosed ITP with SRL and/or steroids. Another limitation of this study is that the bleeding score and health-related quality of life (QOL) were not evaluated during follow-up. However, it has been reported that SRL-based therapy for kidney transplant patients resulted in less fatigue, and better vitality (29, 30).

In conclusion, to the knowledge of our clinical team, this is the largest prospective study to date evaluating SRL as a rescue therapy in patients with R/R ITP. The efficacy of SRL in these patients is superior to rituximab and Eltrompobag, while sparing severe side-effects. In addition to splenectomy, SRL is a potential therapeutic alternative for patients with R/R ITP. Of course, further standard multi-center RCTs will be needed to test application of SRL as a first line treatment for ITP. Taken together, we propose that SRL promotes and can re-establish peripheral tolerance through the regulation of T, B, and myeloid cell subsets in ITP patients; additional studies will explore this mechanism in more depth.

## Data Availability Statement

All datasets generated for this study are included in the article/supplementary material.

## Ethics Statement

The studies involving human participants were reviewed and approved by the ethics committees of Xinqiao Hospital. Written informed consent to participate in this study was provided by the participants' legal guardian/next of kin. Written informed consent was obtained from the individual(s), and minor(s)' legal guardian/next of kin, for the publication of any potentially identifiable images or data included in this article.

## Author Contributions

YF contributed to the design and conceptualization of the research, design of data analyses, interpretation of data, collection of data, and writing of the manuscript. HY, LeG, CZ, YL, PK, and LiG participated in patient recruitment. YX, PW, WZ, KC, ZZ, TC, YQ, SY, RW, XL, KW, and ZW contributed to the collection of data and samples, FACS analysis and revision of the manuscript. XZ contributed to the design and conceptualization of the research, coordination of the research as lead investigator, and revision of the manuscript. All authors reviewed the manuscript and approved the final version for submission.

### Conflict of Interest

The authors declare that the research was conducted in the absence of any commercial or financial relationships that could be construed as a potential conflict of interest.

## References

[1] Cuker A Neunert CE. How I treat refractory immune thrombocytopenia. Blood. (2016) 128:1547–54. 10.1182/blood-2016-03-603365 27053529

[2] Li X Gao Q Feng Y Zhang X. Developing role of B cells in the pathogenesis and treatment of chronic GVHD. Br J Haematol. (2019) 184:323–36. 10.1111/bjh.15719 30585319 PMC6590173

[3] Weichhart T. mTOR as Regulator of Lifespan, aging, and cellular senescence: a mini-review. Gerontology. (2018) 64:127–34. 10.1159/000484629 29190625 PMC6089343

[4] Villa A Aboalela A Luskin KA Cutler CS Sonis ST Woo SB. Mammalian target of rapamycin inhibitor-associated stomatitis in hematopoietic stem cell transplantation patients receiving sirolimus prophylaxis for graft-versus-host disease. Biol Blood Marrow Transplant. (2015) 21:503–8. 10.1016/j.bbmt.2014.11.680 25482865

[5] Teachey DT Greiner R Seif A Attiyeh E Bleesing J Choi J. Treatment with sirolimus results in complete responses in patients with autoimmune lymphoproliferative syndrome. Br J Haematol. (2009) 145:101–6. 10.1111/j.1365-2141.2009.07595.x 19208097 PMC2819393

[6] Bride KL Vincent T Smith-Whitley K Lambert MP Bleesing JJ Seif AE. Sirolimus is effective in relapsed/refractory autoimmune cytopenias: results of a prospective multi-institutional trial. Blood. (2016) 127:17–28. 10.1182/blood-2015-07-657981 26504182 PMC4705607

[7] Huang N Perl A. Metabolism as a target for modulation in autoimmune diseases. Trends Immunol. (2018) 39:562–76. 10.1016/j.it.2018.04.006 29739666

[8] Perl A. mTOR-dependent autophagy contributes to end-organ resistance and serves as target for treatment in autoimmune disease. EBioMedicine. (2018) 36:12–3. 10.1016/j.ebiom.2018.09.033 30262258 PMC6197622

[9] Perl A. mTOR activation is a biomarker and a central pathway to autoimmune disorders, cancer, obesity, and aging. Ann N Y Acad Sci. (2015) 1346:33–44. 10.1111/nyas.12756 25907074 PMC4480196

[10] Sun RJ Shan NN. Megakaryocytic dysfunction in immune thrombocytopenia is linked to autophagy. Cancer Cell Int. (2019) 19:59. 10.1186/s12935-019-0779-0 30923461 PMC6419848

[11] Lai ZW Kelly R Winans T Marchena I Shadakshari A Yu J. Sirolimus in patients with clinically active systemic lupus erythematosus resistant to, or intolerant of, conventional medications: a single-arm, open-label, phase 1/2 trial. Lancet. (2018) 391:1186–96. 10.1016/S0140-6736(18)30485-9 29551338 PMC5891154

[12] Jasinski S Weinblatt ME Glasser CL. Sirolimus as an effective agent in the treatment of immune thrombocytopenia (ITP) and evans syndrome (ES): a single institution's experience. J Pediatr Hematol Oncol. (2017) 39:420–4. 10.1097/MPH.0000000000000818 28267088

[13] Liu XG Bai XC Chen FP Cheng YF Dai KS Fang MY. Chinese guidelines for treatment of adult primary immune thrombocytopenia. Int J Hematol. (2018) 107:615–23. 10.1007/s12185-018-2445-z 29619624

[14] Long Z Yu F Du Y Li H Chen M Zhuang J. Successful treatment of refractory/relapsed acquired pure red cell aplasia with sirolimus. Ann Hematol. (2018) 97:2047–54. 10.1007/s00277-018-3431-5 29982851

[15] Montalvo J Velazquez D Pantoja JP Sierra M López-Karpovitch X Herrera MF. Laparoscopic splenectomy for primary immune thrombocytopenia: clinical outcome and prognostic factors. J Laparoendosc Adv Surg Tech A. (2014) 24:466–70. 10.1089/lap.2013.0267 24905792

[16] Rodeghiero F Ruggeri M. Is splenectomy still the gold standard for the treatment of chronic ITP? Am J Hematol. (2008) 83:91. 10.1002/ajh.21109 17994550

[17] Tran H Brighton T Grigg A McRae S Dixon J Thurley D. A multi-centre, single-arm, open-label study evaluating the safety and efficacy of fixed dose rituximab in patients with refractory, relapsed or chronic idiopathic thrombocytopenic purpura (R-ITP1000 study). Br J Haematol. (2014) 167:243–51. 10.1111/bjh.13029 25041261

[18] Cooper N Arnold DM. The effect of rituximab on humoral and cell mediated immunity and infection in the treatment of autoimmune diseases. Br J Haematol. (2010) 149:3–13. 10.1111/j.1365-2141.2010.08076.x 20151975

[19] Cheng G Saleh MN Marcher C Vasey S Mayer B Aivado M. Eltrombopag for management of chronic immune thrombocytopenia (RAISE): a 6-month, randomised, phase 3 study. Lancet. (2011) 377:393–402. 10.1016/S0140-6736(10)60959-2 20739054

[20] Yang R Li J Jin J Huang M Yu Z Xu X. Multicentre, randomised phase III study of the efficacy and safety of eltrombopag in Chinese patients with chronic immune thrombocytopenia. Br J Haematol. (2017) 176:101–10. 10.1111/bjh.14380 27734464

[21] Sandmaier BM Kornblit B Storer BE Olesen G Maris MB Langston AA. Addition of sirolimus to standard cyclosporine plus mycophenolate mofetil-based graft-versus-host disease prophylaxis for patients after unrelated non-myeloablative haemopoietic stem cell transplantation: a multicentre, randomised, phase 3 trial. Lancet Haematol. (2019) 6:E409–18. 10.1016/S2352-3026(19)30088-2 31248843 PMC6686903

[22] Gooptu M Kim HT Howard A Choi SW Soiffer RJ Antin JH Effect of sirolimus on immune reconstitution following myeloablative allogeneic stem-cell transplantation: a *post-hoc* analysis of a randomized controlled trial comparing sirolimus/tacrolimus with tacrolimus/methotrexate (BMT CTN 0402). Blood. (2018) 132(Suppl 1):2110 10.1182/blood-2018-99-11566230442746

[23] Li J Wang Z Dai L Cao L Su J Zhu M. Effects of rapamycin combined with low dose prednisone in patients with chronic immune thrombocytopenia. Clin Dev Immunol. (2013) 2013:548085. 10.1155/2013/548085 24363761 PMC3865723

[24] McKenzie CG Guo L Freedman J Semple JW. Cellular immune dysfunction in immune thrombocytopenia (ITP). Br J Haematol. (2013) 163:10–23. 10.1111/bjh.12480 23937260

[25] Semple JW Provan D. The immunopathogenesis of immune thrombocytopenia: T cells still take center-stage. Curr Opin Hematol. (2012) 19:357–62. 10.1097/MOH.0b013e3283567541 22759631

[26] Zhang J Ma D Zhu X Qu X Ji C Hou M. Elevated profile of Th17, Th1 and Tc1 cells in patients with immune thrombocytopenic purpura. Haematologica. (2009) 94:1326–9. 10.3324/haematol.2009.007823 19734430 PMC2738732

[27] Mills CD Kincaid K Alt JM Heilman MJ Hill AM. M-1/M-2 macrophages and the Th1/Th2 paradigm. J Immunol. (2000) 164:6166–73. 10.4049/jimmunol.164.12.6166 10843666

[28] Chen YY Zhou YQ Zhao N Zhang Y Xu WQ Tang YM. Evaluation of IVIG response in relation to Th1/Th2 cytokines in pediatricm immune thrombocytopenia. Cytokine. (2019) 120:234–41. 10.1016/j.cyto.2019.05.014 31129375

[29] Oberbauer R Hutchison B Eris J Arias M Claesson K Mota A. Health-related quality-of-life outcomes of sirolimus-treated kidney transplant patients after elimination of cyclosporine A: results of a 2-year randomized clinical trial. Transplantation. (2003) 75:1277–85. 10.1097/01.TP.0000061766.37366.6B 12717216

[30] Russ G Jamieson N Oberbauer R Arias M Murgia MG Blancho G. Three-year health-related quality-of-life outcomes for sirolimus-treated kidney transplant patients after elimination of cyclosporine. Transpl Int. (2007) 20:875–83. 10.1111/j.1432-2277.2007.00547.x 17854445

